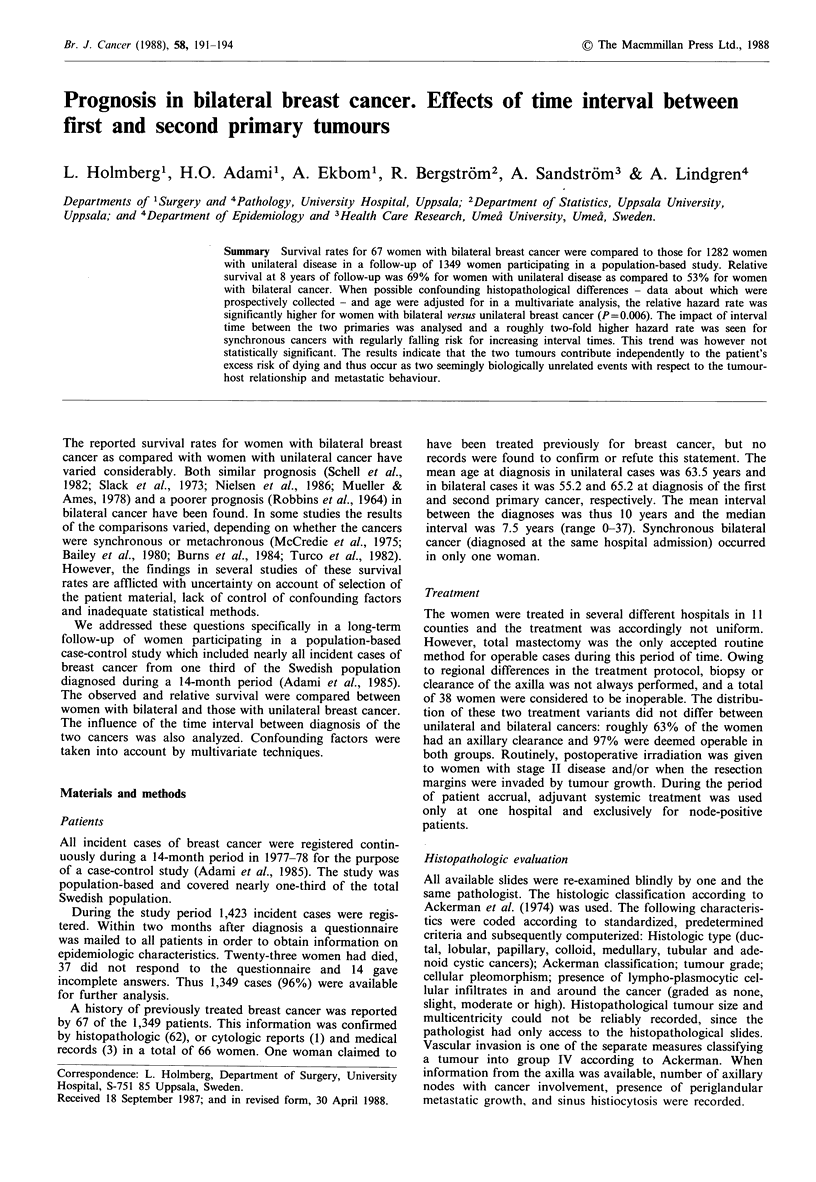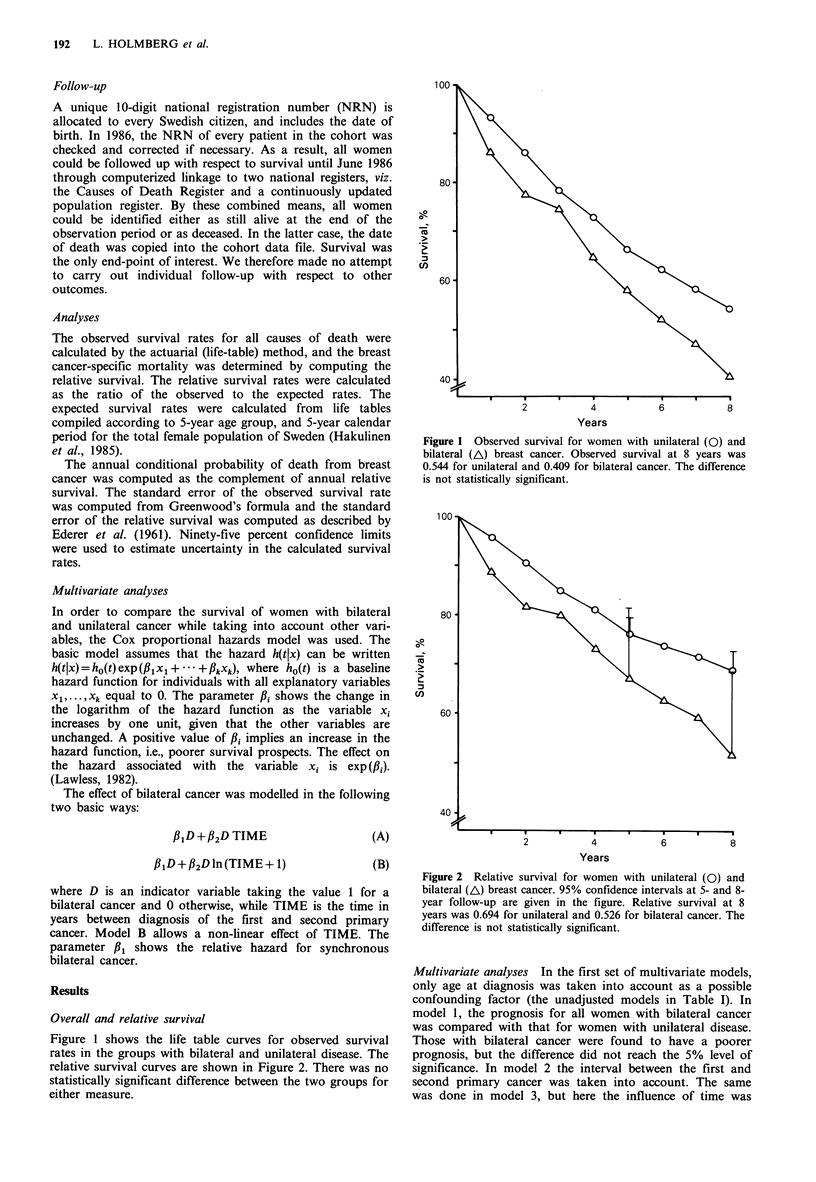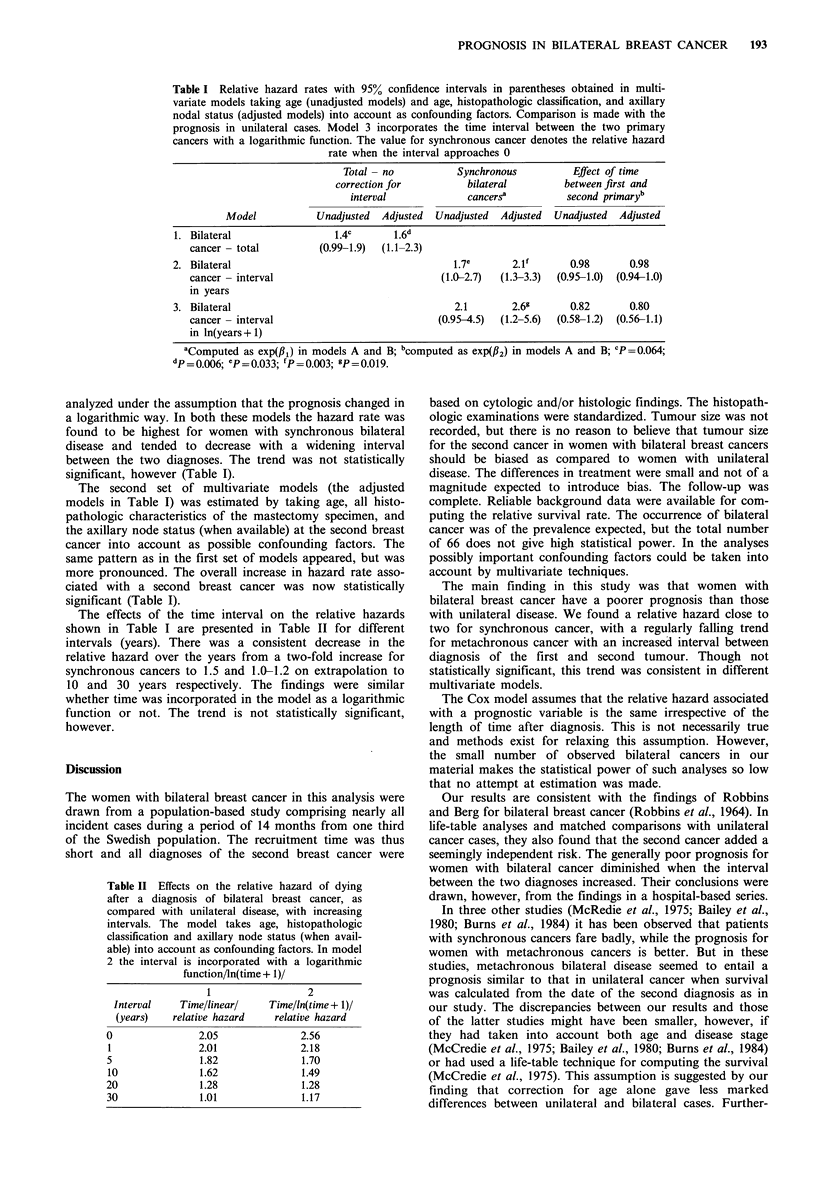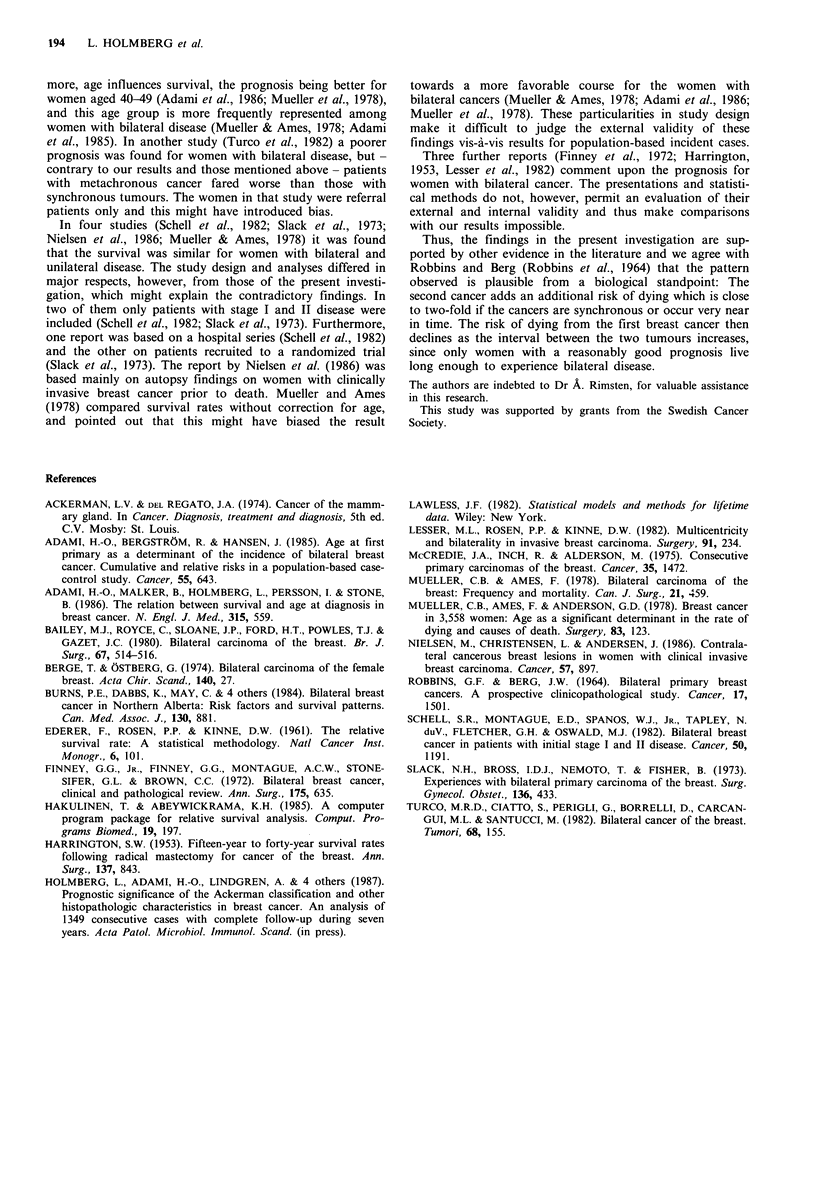# Prognosis in bilateral breast cancer. Effects of time interval between first and second primary tumours.

**DOI:** 10.1038/bjc.1988.191

**Published:** 1988-08

**Authors:** L. Holmberg, H. O. Adami, A. Ekbom, R. BergstrÃ¶m, A. SandstrÃ¶m, A. Lindgren

**Affiliations:** Department of Surgery, University Hospital, Uppsala, Sweden.

## Abstract

Survival rates for 67 women with bilateral breast cancer were compared to those for 1282 women with unilateral disease in a follow-up of 1349 women participating in a population-based study. Relative survival at 8 years of follow-up was 69% for women with unilateral disease as compared to 53% for women with bilateral cancer. When possible confounding histopathological differences--data about which were prospectively collected--and age were adjusted for in a multivariate analysis, the relative hazard rate was significantly higher for women with bilateral versus unilateral breast cancer (P = 0.006). The impact of interval time between the two primaries was analysed and a roughly two-fold higher hazard rate was seen for synchronous cancers with regularly falling risk for increasing interval times. This trend was however not statistically significant. The results indicate that the two tumours contribute independently to the patient's excess risk of dying and thus occur as two seemingly biologically unrelated events with respect to the tumour-host relationship and metastatic behaviour.


Br. J. Cancer (1988), 58, 191-194 © The Macmmillan Press Ltd., 1988

### Prognosis in bilateral breast cancer. Effects of time interval between first and second primary tumours

L. Holmberg[1], H.O. Adami[1], A. Ekbom[1], R. Bergström[2], A. Sandström[3] & A. Lindgren[4]

*Departments of [1]Surgery and [4]Pathology, University Hospital, Uppsala; [2]Department of Statistics, Uppsala University, Uppsala; and [4]Department of Epidemiology and [3]Health Care Research, Umeå University, Umeå, Sweden.*

**Summary** Survival rates for 67 women with bilateral breast cancer were compared to those for 1282 women with unilateral disease in a follow-up of 1349 women participating in a population-based study. Relative survival at 8 years of follow-up was 69% for women with unilateral disease as compared to 53% for women with bilateral cancer. When possible confounding histopathological differences – data about which were prospectively collected – and age were adjusted for in a multivariate analysis, the relative hazard rate was significantly higher for women with bilateral *versus* unilateral breast cancer ($P=0.006$). The impact of interval time between the two primaries was analysed and a roughly two-fold higher hazard rate was seen for synchronous cancers with regularly falling risk for increasing interval times. This trend was however not statistically significant. The results indicate that the two tumours contribute independently to the patient's excess risk of dying and thus occur as two seemingly biologically unrelated events with respect to the tumour-host relationship and metastatic behaviour.

The reported survival rates for women with bilateral breast cancer as compared with women with unilateral cancer have varied considerably. Both similar prognosis (Schell *et al.*, 1982; Slack *et al.*, 1973; Nielsen *et al.*, 1986; Mueller & Ames, 1978) and a poorer prognosis (Robbins *et al.*, 1964) in bilateral cancer have been found. In some studies the results of the comparisons varied, depending on whether the cancers were synchronous or metachronous (McCredie *et al.*, 1975; Bailey *et al.*, 1980; Burns *et al.*, 1984; Turco *et al.*, 1982). However, the findings in several studies of these survival rates are afflicted with uncertainty on account of selection of the patient material, lack of control of confounding factors and inadequate statistical methods.

We addressed these questions specifically in a long-term follow-up of women participating in a population-based case-control study which included nearly all incident cases of breast cancer from one third of the Swedish population diagnosed during a 14-month period (Adami *et al.*, 1985). The observed and relative survival were compared between women with bilateral and those with unilateral breast cancer. The influence of the time interval between diagnosis of the two cancers was also analyzed. Confounding factors were taken into account by multivariate techniques.

### Materials and methods

### *Patients*

All incident cases of breast cancer were registered continuously during a 14-month period in 1977–78 for the purpose of a case-control study (Adami *et al.*, 1985). The study was population-based and covered nearly one-third of the total Swedish population.

During the study period 1,423 incident cases were registered. Within two months after diagnosis a questionnaire was mailed to all patients in order to obtain information on epidemiologic characteristics. Twenty-three women had died, 37 did not respond to the questionnaire and 14 gave incomplete answers. Thus 1,349 cases (96%) were available for further analysis.

A history of previously treated breast cancer was reported by 67 of the 1,349 patients. This information was confirmed by histopathologic (62), or cytologic reports (1) and medical records (3) in a total of 66 women. One woman claimed to have been treated previously for breast cancer, but no records were found to confirm or refute this statement. The mean age at diagnosis in unilateral cases was 63.5 years and in bilateral cases it was 55.2 and 65.2 at diagnosis of the first and second primary cancer, respectively. The mean interval between the diagnoses was thus 10 years and the median interval was 7.5 years (range 0–37). Synchronous bilateral cancer (diagnosed at the same hospital admission) occurred in only one woman.

Correspondence: L. Holmberg, Department of Surgery, University Hospital, S-751 85 Uppsala, Sweden.
Received 18 September 1987; and in revised form, 30 April 1988.

### *Treatment*

The women were treated in several different hospitals in 11 counties and the treatment was accordingly not uniform. However, total mastectomy was the only accepted routine method for operable cases during this period of time. Owing to regional differences in the treatment protocol, biopsy or clearance of the axilla was not always performed, and a total of 38 women were considered to be inoperable. The distribution of these two treatment variants did not differ between unilateral and bilateral cancers: roughly 63% of the women had an axillary clearance and 97% were deemed operable in both groups. Routinely, postoperative irradiation was given to women with stage II disease and/or when the resection margins were invaded by tumour growth. During the period of patient accrual, adjuvant systemic treatment was used only at one hospital and exclusively for node-positive patients.

### *Histopathologic evaluation*

All available slides were re-examined blindly by one and the same pathologist. The histologic classification according to Ackerman *et al.* (1974) was used. The following characteristics were coded according to standardized, predetermined criteria and subsequently computerized: Histologic type (ductal, lobular, papillary, colloid, medullary, tubular and adenoid cystic cancers); Ackerman classification; tumour grade; cellular pleomorphism; presence of lympho-plasmocytic cellular infiltrates in and around the cancer (graded as none, slight, moderate or high). Histopathological tumour size and multicentricity could not be reliably recorded, since the pathologist had only access to the histopathological slides. Vascular invasion is one of the separate measures classifying a tumour into group IV according to Ackerman. When information from the axilla was available, number of axillary nodes with cancer involvement, presence of periglandular metastatic growth, and sinus histiocytosis were recorded.

*Follow-up*

A unique 10-digit national registration number (NRN) is allocated to every Swedish citizen, and includes the date of birth. In 1986, the NRN of every patient in the cohort was checked and corrected if necessary. As a result, all women could be followed up with respect to survival until June 1986 through computerized linkage to two national registers, *viz.* the Causes of Death Register and a continuously updated population register. By these combined means, all women could be identified either as still alive at the end of the observation period or as deceased. In the latter case, the date of death was copied into the cohort data file. Survival was the only end-point of interest. We therefore made no attempt to carry out individual follow-up with respect to other outcomes.

*Analyses*

The observed survival rates for all causes of death were calculated by the actuarial (life-table) method, and the breast cancer-specific mortality was determined by computing the relative survival. The relative survival rates were calculated as the ratio of the observed to the expected rates. The expected survival rates were calculated from life tables compiled according to 5-year age group, and 5-year calendar period for the total female population of Sweden (Hakulinen *et al.*, 1985).

The annual conditional probability of death from breast cancer was computed as the complement of annual relative survival. The standard error of the observed survival rate was computed from Greenwood's formula and the standard error of the relative survival was computed as described by Ederer *et al.* (1961). Ninety-five percent confidence limits were used to estimate uncertainty in the calculated survival rates.

*Multivariate analyses*

In order to compare the survival of women with bilateral and unilateral cancer while taking into account other variables, the Cox proportional hazards model was used. The basic model assumes that the hazard $h(t|x)$ can be written $h(t|x)=h_0(t)\exp(\beta_1 x_1+\cdots+\beta_k x_k)$, where $h_0(t)$ is a baseline hazard function for individuals with all explanatory variables $x_1,\ldots,x_k$ equal to 0. The parameter $\beta_i$ shows the change in the logarithm of the hazard function as the variable $x_i$ increases by one unit, given that the other variables are unchanged. A positive value of $\beta_i$ implies an increase in the hazard function, i.e., poorer survival prospects. The effect on the hazard associated with the variable $x_i$ is $\exp(\beta_i)$. (Lawless, 1982).

The effect of bilateral cancer was modelled in the following two basic ways:

$$\beta_1 D+\beta_2 D\,\text{TIME} \qquad \text{(A)}$$

$$\beta_1 D+\beta_2 D\ln(\text{TIME}+1) \qquad \text{(B)}$$

where $D$ is an indicator variable taking the value 1 for a bilateral cancer and 0 otherwise, while TIME is the time in years between diagnosis of the first and second primary cancer. Model B allows a non-linear effect of TIME. The parameter $\beta_1$ shows the relative hazard for synchronous bilateral cancer.

### Results

*Overall and relative survival*

Figure 1 shows the life table curves for observed survival rates in the groups with bilateral and unilateral disease. The relative survival curves are shown in Figure 2. There was no statistically significant difference between the two groups for either measure.

**Figure 1** Observed survival for women with unilateral (○) and bilateral (△) breast cancer. Observed survival at 8 years was 0.544 for unilateral and 0.409 for bilateral cancer. The difference is not statistically significant.

**Figure 2** Relative survival for women with unilateral (○) and bilateral (△) breast cancer. 95% confidence intervals at 5- and 8-year follow-up are given in the figure. Relative survival at 8 years was 0.694 for unilateral and 0.526 for bilateral cancer. The difference is not statistically significant.

*Multivariate analyses* In the first set of multivariate models, only age at diagnosis was taken into account as a possible confounding factor (the unadjusted models in Table I). In model 1, the prognosis for all women with bilateral cancer was compared with that for women with unilateral disease. Those with bilateral cancer were found to have a poorer prognosis, but the difference did not reach the 5% level of significance. In model 2 the interval between the first and second primary cancer was taken into account. The same was done in model 3, but here the influence of time was

**Table I** Relative hazard rates with 95% confidence intervals in parentheses obtained in multivariate models taking age (unadjusted models) and age, histopathologic classification, and axillary nodal status (adjusted models) into account as confounding factors. Comparison is made with the prognosis in unilateral cases. Model 3 incorporates the time interval between the two primary cancers with a logarithmic function. The value for synchronous cancer denotes the relative hazard rate when the interval approaches 0

| | *Total – no correction for interval* | | *Synchronous bilateral cancers*[a] | | *Effect of time between first and second primary*[b] | |
|---|---|---|---|---|---|---|
| *Model* | *Unadjusted* | *Adjusted* | *Unadjusted* | *Adjusted* | *Unadjusted* | *Adjusted* |
| 1. Bilateral cancer – total | 1.4[c] (0.99–1.9) | 1.6[d] (1.1–2.3) | | | | |
| 2. Bilateral cancer – interval in years | | | 1.7[e] (1.0–2.7) | 2.1[f] (1.3–3.3) | 0.98 (0.95–1.0) | 0.98 (0.94–1.0) |
| 3. Bilateral cancer – interval in ln(years + 1) | | | 2.1 (0.95–4.5) | 2.6[g] (1.2–5.6) | 0.82 (0.58–1.2) | 0.80 (0.56–1.1) |

[a]Computed as $\exp(\beta_1)$ in models A and B; [b]computed as $\exp(\beta_2)$ in models A and B; [c]$P=0.064$; [d]$P=0.006$; [e]$P=0.033$; [f]$P=0.003$; [g]$P=0.019$.

analyzed under the assumption that the prognosis changed in a logarithmic way. In both these models the hazard rate was found to be highest for women with synchronous bilateral disease and tended to decrease with a widening interval between the two diagnoses. The trend was not statistically significant, however (Table I).

The second set of multivariate models (the adjusted models in Table I) was estimated by taking age, all histopathologic characteristics of the mastectomy specimen, and the axillary node status (when available) at the second breast cancer into account as possible confounding factors. The same pattern as in the first set of models appeared, but was more pronounced. The overall increase in hazard rate associated with a second breast cancer was now statistically significant (Table I).

The effects of the time interval on the relative hazards shown in Table I are presented in Table II for different intervals (years). There was a consistent decrease in the relative hazard over the years from a two-fold increase for synchronous cancers to 1.5 and 1.0–1.2 on extrapolation to 10 and 30 years respectively. The findings were similar whether time was incorporated in the model as a logarithmic function or not. The trend is not statistically significant, however.

### Discussion

The women with bilateral breast cancer in this analysis were drawn from a population-based study comprising nearly all incident cases during a period of 14 months from one third of the Swedish population. The recruitment time was thus short and all diagnoses of the second breast cancer were based on cytologic and/or histologic findings. The histopathologic examinations were standardized. Tumour size was not recorded, but there is no reason to believe that tumour size for the second cancer in women with bilateral breast cancers should be biased as compared to women with unilateral disease. The differences in treatment were small and not of a magnitude expected to introduce bias. The follow-up was complete. Reliable background data were available for computing the relative survival rate. The occurrence of bilateral cancer was of the prevalence expected, but the total number of 66 does not give high statistical power. In the analyses possibly important confounding factors could be taken into account by multivariate techniques.

The main finding in this study was that women with bilateral breast cancer have a poorer prognosis than those with unilateral disease. We found a relative hazard close to two for synchronous cancer, with a regularly falling trend for metachronous cancer with an increased interval between diagnosis of the first and second tumour. Though not statistically significant, this trend was consistent in different multivariate models.

The Cox model assumes that the relative hazard associated with a prognostic variable is the same irrespective of the length of time after diagnosis. This is not necessarily true and methods exist for relaxing this assumption. However, the small number of observed bilateral cancers in our material makes the statistical power of such analyses so low that no attempt at estimation was made.

Our results are consistent with the findings of Robbins and Berg for bilateral breast cancer (Robbins *et al.*, 1964). In life-table analyses and matched comparisons with unilateral cancer cases, they also found that the second cancer added a seemingly independent risk. The generally poor prognosis for women with bilateral cancer diminished when the interval between the two diagnoses increased. Their conclusions were drawn, however, from the findings in a hospital-based series.

In three other studies (McRedie *et al.*, 1975; Bailey *et al.*, 1980; Burns *et al.*, 1984) it has been observed that patients with synchronous cancers fare badly, while the prognosis for women with metachronous cancers is better. But in these studies, metachronous bilateral disease seemed to entail a prognosis similar to that in unilateral cancer when survival was calculated from the date of the second diagnosis as in our study. The discrepancies between our results and those of the latter studies might have been smaller, however, if they had taken into account both age and disease stage (McCredie *et al.*, 1975; Bailey *et al.*, 1980; Burns *et al.*, 1984) or had used a life-table technique for computing the survival (McCredie *et al.*, 1975). This assumption is suggested by our finding that correction for age alone gave less marked differences between unilateral and bilateral cases. Further-

**Table II** Effects on the relative hazard of dying after a diagnosis of bilateral breast cancer, as compared with unilateral disease, with increasing intervals. The model takes age, histopathologic classification and axillary node status (when available) into account as confounding factors. In model 2 the interval is incorporated with a logarithmic function/ln(time + 1)/

| *Interval (years)* | 1 *Time/linear/ relative hazard* | 2 *Time/ln(time + 1)/ relative hazard* |
|---|---|---|
| 0 | 2.05 | 2.56 |
| 1 | 2.01 | 2.18 |
| 5 | 1.82 | 1.70 |
| 10 | 1.62 | 1.49 |
| 20 | 1.28 | 1.28 |
| 30 | 1.01 | 1.17 |

more, age influences survival, the prognosis being better for women aged 40–49 (Adami *et al.*, 1986; Mueller *et al.*, 1978), and this age group is more frequently represented among women with bilateral disease (Mueller & Ames, 1978; Adami *et al.*, 1985). In another study (Turco *et al.*, 1982) a poorer prognosis was found for women with bilateral disease, but – contrary to our results and those mentioned above – patients with metachronous cancer fared worse than those with synchronous tumours. The women in that study were referral patients only and this might have introduced bias.

In four studies (Schell *et al.*, 1982; Slack *et al.*, 1973; Nielsen *et al.*, 1986; Mueller & Ames, 1978) it was found that the survival was similar for women with bilateral and unilateral disease. The study design and analyses differed in major respects, however, from those of the present investigation, which might explain the contradictory findings. In two of them only patients with stage I and II disease were included (Schell *et al.*, 1982; Slack *et al.*, 1973). Furthermore, one report was based on a hospital series (Schell *et al.*, 1982) and the other on patients recruited to a randomized trial (Slack *et al.*, 1973). The report by Nielsen *et al.* (1986) was based mainly on autopsy findings on women with clinically invasive breast cancer prior to death. Mueller and Ames (1978) compared survival rates without correction for age, and pointed out that this might have biased the result towards a more favorable course for the women with bilateral cancers (Mueller & Ames, 1978; Adami *et al.*, 1986; Mueller *et al.*, 1978). These particularities in study design make it difficult to judge the external validity of these findings vis-à-vis results for population-based incident cases.

Three further reports (Finney *et al.*, 1972; Harrington, 1953, Lesser *et al.*, 1982) comment upon the prognosis for women with bilateral cancer. The presentations and statistical methods do not, however, permit an evaluation of their external and internal validity and thus make comparisons with our results impossible.

Thus, the findings in the present investigation are supported by other evidence in the literature and we agree with Robbins and Berg (Robbins *et al.*, 1964) that the pattern observed is plausible from a biological standpoint: The second cancer adds an additional risk of dying which is close to two-fold if the cancers are synchronous or occur very near in time. The risk of dying from the first breast cancer then declines as the interval between the two tumours increases, since only women with a reasonably good prognosis live long enough to experience bilateral disease.

The authors are indebted to Dr Å. Rimsten, for valuable assistance in this research.

This study was supported by grants from the Swedish Cancer Society.

### References

ACKERMAN, L.V. & DEL REGATO, J.A. (1974). Cancer of the mammary gland. In *Cancer. Diagnosis, treatment and diagnosis*, 5th ed. C.V. Mosby: St. Louis.

ADAMI, H.-O., BERGSTRÖM, R. & HANSEN, J. (1985). Age at first primary as a determinant of the incidence of bilateral breast cancer. Cumulative and relative risks in a population-based case-control study. *Cancer*, **55**, 643.

ADAMI, H.-O., MALKER, B., HOLMBERG, L., PERSSON, I. & STONE, B. (1986). The relation between survival and age at diagnosis in breast cancer. *N. Engl. J. Med.*, **315**, 559.

BAILEY, M.J., ROYCE, C., SLOANE, J.P., FORD, H.T., POWLES, T.J. & GAZET, J.C. (1980). Bilateral carcinoma of the breast. *Br. J. Surg.*, **67**, 514–516.

BERGE, T. & ÖSTBERG, G. (1974). Bilateral carcinoma of the female breast. *Acta Chir. Scand.*, **140**, 27.

BURNS, P.E., DABBS, K., MAY, C. & 4 others (1984). Bilateral breast cancer in Northern Alberta: Risk factors and survival patterns. *Can. Med. Assoc. J.*, **130**, 881.

EDERER, F., ROSEN, P.P. & KINNE, D.W. (1961). The relative survival rate: A statistical methodology. *Natl Cancer Inst. Monogr.*, **6**, 101.

FINNEY, G.G., JR., FINNEY, G.G., MONTAGUE, A.C.W., STONESIFER, G.L. & BROWN, C.C. (1972). Bilateral breast cancer, clinical and pathological review. *Ann. Surg.*, **175**, 635.

HAKULINEN, T. & ABEYWICKRAMA, K.H. (1985). A computer program package for relative survival analysis. *Comput. Programs Biomed.*, **19**, 197.

HARRINGTON, S.W. (1953). Fifteen-year to forty-year survival rates following radical mastectomy for cancer of the breast. *Ann. Surg.*, **137**, 843.

HOLMBERG, L., ADAMI, H.-O., LINDGREN, A. & 4 others (1987). Prognostic significance of the Ackerman classification and other histopathologic characteristics in breast cancer. An analysis of 1349 consecutive cases with complete follow-up during seven years. *Acta Patol. Microbiol. Immunol. Scand.* (in press).

LAWLESS, J.F. (1982). *Statistical models and methods for lifetime data.* Wiley: New York.

LESSER, M.L., ROSEN, P.P. & KINNE, D.W. (1982). Multicentricity and bilaterality in invasive breast carcinoma. *Surgery*, **91**, 234.

MCCREDIE, J.A., INCH, R. & ALDERSON, M. (1975). Consecutive primary carcinomas of the breast. *Cancer*, **35**, 1472.

MUELLER, C.B. & AMES, F. (1978). Bilateral carcinoma of the breast: Frequency and mortality. *Can. J. Surg.*, **21**, 459.

MUELLER, C.B., AMES, F. & ANDERSON, G.D. (1978). Breast cancer in 3,558 women: Age as a significant determinant in the rate of dying and causes of death. *Surgery*, **83**, 123.

NIELSEN, M., CHRISTENSEN, L. & ANDERSEN, J. (1986). Contralateral cancerous breast lesions in women with clinical invasive breast carcinoma. *Cancer*, **57**, 897.

ROBBINS, G.F. & BERG, J.W. (1964). Bilateral primary breast cancers. A prospective clinicopathological study. *Cancer*, **17**, 1501.

SCHELL, S.R., MONTAGUE, E.D., SPANOS, W.J., JR., TAPLEY, N. duV., FLETCHER, G.H. & OSWALD, M.J. (1982). Bilateral breast cancer in patients with initial stage I and II disease. *Cancer*, **50**, 1191.

SLACK, N.H., BROSS, I.D.J., NEMOTO, T. & FISHER, B. (1973). Experiences with bilateral primary carcinoma of the breast. *Surg. Gynecol. Obstet.*, **136**, 433.

TURCO, M.R.D., CIATTO, S., PERIGLI, G., BORRELLI, D., CARCANGUI, M.L. & SANTUCCI, M. (1982). Bilateral cancer of the breast. *Tumori*, **68**, 155.